# Reassortant Eurasian Avian-Like Influenza A(H1N1) Virus from a Severely Ill Child, Hunan Province, China, 2015

**DOI:** 10.3201/eid2211.160181

**Published:** 2016-11

**Authors:** Wenfei Zhu, Hong Zhang, Xingyu Xiang, Lili Zhong, Lei Yang, Junfeng Guo, Yiran Xie, Fangcai Li, Zhihong Deng, Hong Feng, Yiwei Huang, Shixiong Hu, Xin Xu, Xiaohui Zou, Xiaodan Li, Tian Bai, Yongkun Chen, Zi Li, Junhua Li, Yuelong Shu

**Affiliations:** National Institute for Viral Disease Control and Prevention, Chinese Center for Disease Control and Prevention, Beijing, China (W. Zhu, L. Yang, J. Guo, Y. Xie, X. Zou, X. Li, T. Bai, Y. Chen, Z. Li, Y. Shu);; Key Laboratory for Medical Virology, National Health and Family Planning Commission, Beijing (W. Zhu, L. Yang, J. Guo, Y. Xie, X. Zou, X. Li, T. Bai, Y. Chen, Z. Li, Y. Shu);; Hunan Provincial Center for Disease Control and Prevention, Changsha, China (H. Zhang, X. Xiang, F. Li, Z. Deng, Y. Huang, S. Hu, J. Li);; Hunan Provincial People’s Hospital, Changsha (L. Zhong); Liuyang Center for Disease Control and Prevention, Liuyang, China (H. Feng, X. Xu)

**Keywords:** viruses, influenza virus, influenza A(H1N1) virus, influenza, reassortant virus, genetic reassortant, Eurasian avian-like virus, EA-H1N1, clinical features, infectivity, virulence, China, novel virus, human origin, swine influenza virus, respiratory infections

## Abstract

In 2015, a novel influenza A(H1N1) virus was isolated from a boy in China who had severe pneumonia. The virus was a genetic reassortant of Eurasian avian-like influenza A(H1N1) (EA-H1N1) virus. The hemagglutinin, neuraminidase, and matrix genes of the reassortant virus were highly similar to genes in EA-H1N1 swine influenza viruses, the polybasic 1 and 2, polymerase acidic, and nucleoprotein genes originated from influenza A(H1N1)pdm09 virus, and the nonstructural protein gene derived from classical swine influenza A(H1N1) (CS H1N1) virus. In a mouse model, the reassortant virus, termed influenza A/Hunan/42443/2015(H1N1) virus, showed higher infectivity and virulence than another human EA-H1N1 isolate, influenza A/Jiangsu/1/2011(H1N1) virus. In the respiratory tract of mice, virus replication by influenza A/Hunan/42443/2015(H1N1) virus was substantially higher than that by influenza A/Jiangsu/1/2011(H1N1) virus. Human-to-human transmission of influenza A/Hunan/42443/2015(H1N1) virus has not been detected; however, given the circulation of novel EA-H1N1 viruses in pigs, enhanced surveillance should be instituted among swine and humans.

## Abstract 2

Infectivity and virulence of this virus in mice are higher than for previous human-origin Eurasian avian–like viruses.

Pigs are well known as genetic mixing vessels for human and avian influenza viruses (*1*,*2*), and swine influenza viruses (SIVs) occasionally infect humans (*3*–*5*). SIV was first reported in humans in 1958 in Czechoslovakia (*6*). The largest outbreak of classical swine influenza A(H1N1) (CS H1N1) virus occurred in Fort Dix, New Jersey, USA, in 1976 (*7*,*8*). Human infections with variant influenza subtype H1N1 and H3N2 viruses with matrix (M) genes derived from swine-origin influenza A(H1N1)pdm09 virus have occurred continuously since the virus was first detected in 2009, and the number of infections has increased substantially in recent years (*6*). Two cases of Eurasian avian-like influenza A(H1N1) (EA-H1N1) infection have been reported in mainland China. The first case, which began in late December 2010 in a 3-year-old boy in Jiangsu Province, resulted in death; however, the child had a history of renal disease (*9*,*10*). The second case, which began in December 2012 in a 3-year-old boy in Hebei Province, caused mild influenza-like illness (*11*).

EA-H1N1 SIVs have been shown to preferentially bind to human-type receptors, and ferrets have been experimentally infected with some EA-H1N1 SIVs via respiratory droplet transmission (*12*). EA-H1N1 SIVs reportedly have the potential to transmit efficiently and cause a pandemic among humans after long-term evolution in pigs (*12*). We report a severe human infection with a reassortant influenza virus in China and the results of genetic, infectivity, and virulence investigations of the novel virus.

## Materials and Methods

### Case Investigation

On June 30, 2015, a 30-month-old boy was admitted to a hospital in Changsha City, Hunan Province, China. The following data were recorded: demographic characteristics; underlying medical conditions; clinical signs, symptoms and complications; chest radiograph findings; laboratory test results; antimicrobial drug treatment; and clinical outcomes.

### Virus Isolation and Titration

We obtained a bronchoalveolar lavage sample from the patient and inoculated it onto MDCK cells for 72 h at 37°C before conducting influenza virus testing. We used Sanger sequencing to subtype the hemagglutinin (HA)–positive isolate from the patient, and we evaluated the bronchoalveolar lavage sample by using next-generation sequencing as previously described (*13*). For additional studies, we propagated the virus in 9- to 11-day-old embryonated chicken eggs for 48 h at 37°C. The allantoic fluid was then harvested and stored at −80°C until use.

For infectivity and virulence analyses, we used influenza A/Jiangsu/1/2011(H1N1) (JS/1/11 EA-H1N1) virus, which had been previously isolated from a child in China with fatal infection and was stored in the Chinese National Influenza Center, Chinese Center for Disease Control and Prevention, Beijing, China. To propagate JS/1/11 EA-H1N1 virus, we inoculated 9-day-old embryonated pathogen-free chicken eggs with 0.2 mL of virus stock and incubated the eggs for 48 h at 35°C. The allantoic fluid was then harvested and stored at −80°C until use.

We determined virus titrations for JS/1/11 EA-H1N1 virus and the patient’s isolate by using MDCK cells. The 50% tissue culture infectious dose (TCID_50_) was calculated by using the Reed–Muench formula (*14*).

### Genetic Analyses

DNA sequences generated by Sanger sequencing of the patient-derived virus were assembled using DNASTAR (http://www.dnastar.com/). All sequences obtained in this study were submitted to the Global Initiative on Sharing Avian Influenza Data (GISAID) database (http://platform.gisaid.org). Multiple sequence alignments were performed with MUSCLE software (http://www.drive5.com/muscle/) using MEGA6 (http://www.megasoftware.net/). To determine the identity of the patient’s isolate, we compared sequences for the isolate with those for viruses in the GISAID database. For comparison, we selected representative isolates for each of the following H1N1 lineages: classical swine, Eurasian avian-like swine, Eurasian avian, North American avian, A(H1N1)pdm09, and seasonal human influenza viruses. We also included recent swine isolates from China with sequences available in GISAID. Phylogenetic relationships were estimated for each of 8 gene segments by using the maximum-likelihood method with the general time-reversible plus gamma distribution plus invariable site substitution model, which was implemented in MEGA6 with 1,000 bootstrap replications.

### Virulence and Replication Studies in Mice

We used a mouse model to evaluate virulence and replication of the patient-derived virus and JS/1/11 EA-H1N1 virus. All experimental protocols in mice were approved by the Ethics Committee of the National Institute for Viral Disease Control and Prevention, Chinese Center for Disease Control and Prevention (approval no. 201509280027). To determine the virulence of the 2 viruses, we used 8- to 10-week-old female C57BL/6J mice (Vital River Laboratories, Beijing, China). The mice were divided into 3 groups and anesthetized with 0.1 mL of pentobarbital sodium. Two groups then were inoculated intranasally with 10-fold serial dilutions (10^1^–10^6^ TCID_50_ in 50 μL of phosphate-buffered saline [PBS]) of patient-derived virus or JS/1/11 EA-H1N1 virus; a control group was injected with PBS. Bodyweight was measured daily; mice that lost >25% of their original weight were euthanized for humane reasons. At 14 days postinoculation (dpi), we collected blood samples from all surviving mice and separated the serum for antibody testing. Hemagglutination inhibition (HI) assays were performed, according to standard protocols, using 0.5% turkey erythrocytes (*15*,*16*). Serum samples with HI antibody titers of <20 were considered negative.

To determine virus replication in infected mice, we intranasally inoculated all 3 groups of 8- to 10-week-old mice (9 mice/group) with 10^4^ or 10^6^ TCID_50_ of JS/1/11 EA-H1N1 virus, the patient-derived virus, or PBS (control group). At 1, 4, and 7 days dpi, we euthanized 3 mice in each group. Brain, nasal turbinates, trachea, lung, heart, spleen, kidney, liver, and intestines were collected for virus titer determination using the TCID_50_ assay and MDCK cells.

## Results

### The Patient

**Figure 1:**
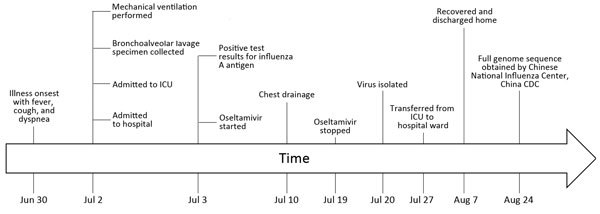
Clinical course for a 30-month-old patient infected with Eurasian avian-like influenza A(H1N1) virus and identification of the causative pathogen, Beijing, China, 2015. China CDC, Chinese Center for Disease Control and Prevention, Beijing; ICU, intensive care unit.

On June 30, 2015, fever (up to 39.5°C), cough, and dyspnea developed in a 30-month-old boy in Hunan Province. On July 2, he was admitted to the intensive care unit of Hunan Provincial People’s Hospital in Changsha, China. Complications (i.e., severe pneumonia, respiratory failure, acute respiratory distress syndrome, and heart failure) were observed (Figure 1). A right pleural effusion and a collapsed lower right lung were noted on a chest radiograph from day 2 after illness onset (Technical Appendix Figure 1). Oseltamivir was administered on July 3, and on July 10, closed drainage of the thoracic cavity was performed. On July 24, day 24 after illness onset, no obvious abnormalities of the cardiac diaphragm were observed (Technical Appendix Figure 1). On July 27, the patient was transferred to the general hospital ward, where he recovered and was discharged on August 7 (Figure 1).

### Virus Isolation and Genetic Analyses

The bronchoalveolar lavage sample from the patient was positive for subtype H1N1 influenza virus based on deep sequencing; no other virus sequences were found (Technical Appendix Figure 2). Virus was isolated from the original patient sample, and Sanger sequencing confirmed the pathogen as a subtype H1N1 influenza virus. We termed the virus influenza A/Hunan/42443/2015(H1N1) (HuN EA-H1N1) and deposited the nucleotide sequences in the GISAID database (accession nos. EPI691392–EPI691399).

Eight genes from HuN EA-H1N1 virus (PB2, PB1, PA, HA, NP, NA, M, and NS) consisted of 2,280, 2,274, 2,151, 1,701, 1,497, 1,410, 982, and 838 nt, respectively. The amino acid motif PSIQSR↓G at the HA cleavage site indicated that the virus was a low pathogenicity influenza virus, and aa 190D and aa 225E (H3 numbering) in the HA protein suggested preferential binding to human influenza virus-binding receptor SAα-2,6-Gal (a sialyl-galactosyl residue with α-2,6-Gal linkage). Residues in the neuraminidase (NA) protein, which were associated with neuraminidase inhibitory drugs (*17*), implied that HuN EA-H1N1 might be sensitive to neuraminidase inhibitors, but an S31N substitution in M2 suggested resistance to M2 ion channel inhibitors.

**Figure 2:**
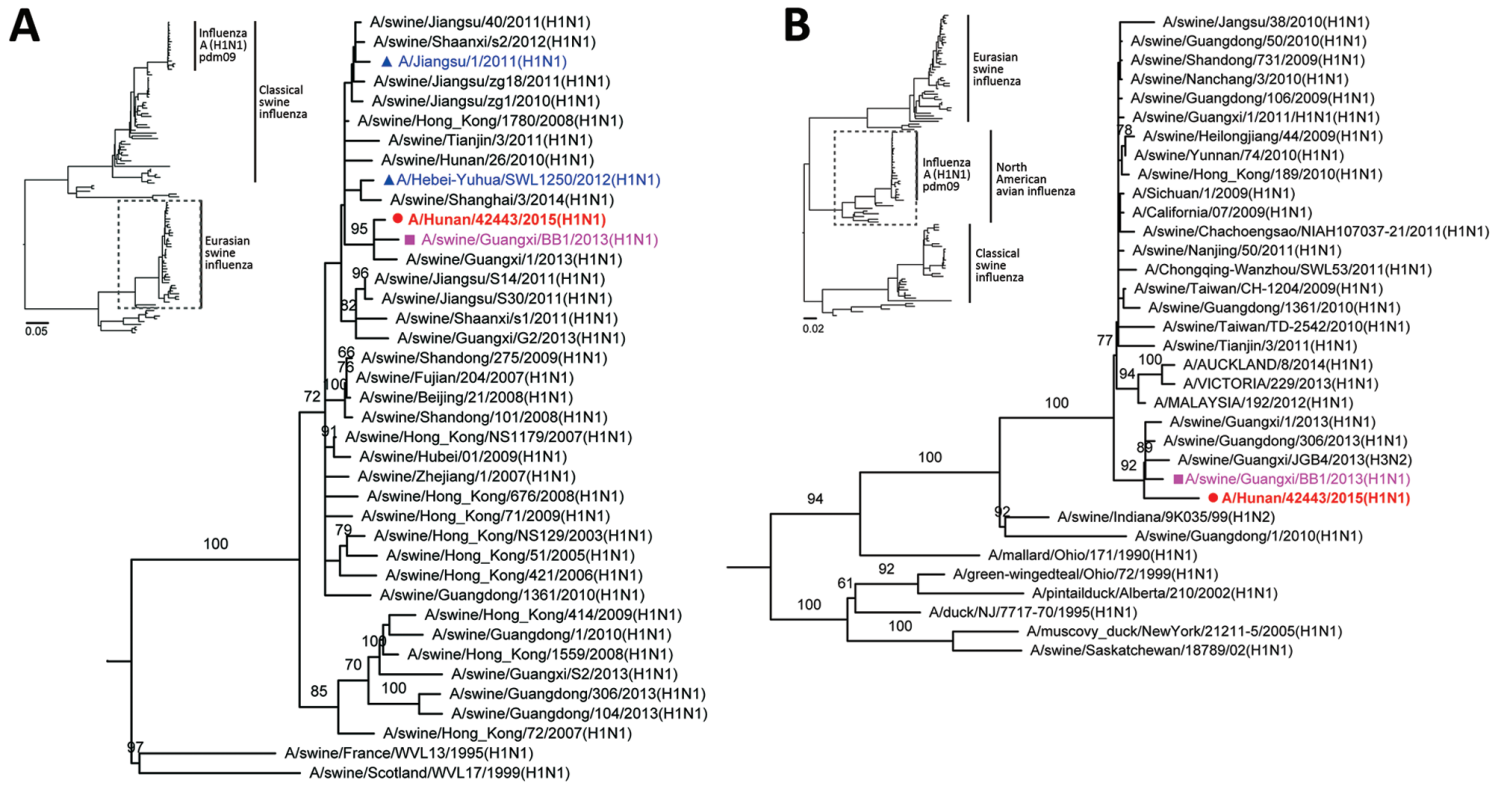
Phylogenetic analysis of Eurasian avian-like influenza A/Hunan/42443/2015 virus (HuN EA-H1N1). A) Analysis of the hemagglutinin gene of representative clades of Eurasian avian-like H1N1 viruses. B) Analysis of the Polymerase basic 2 gene of influenza A(H1N1)pdm09 virus. Insets show evolutionary analyses for all lineages of subtype H1N1 viruses. The reliability of the trees was assessed via bootstrap analysis with 1,000 replications; only bootstrap values >60% are shown. The horizontal distances are proportional to the genetic distance. Red indicates HuN EA-H1N1 virus, the virus reported in this study; pink indicates A/swine/Guangxi/BB1/2013(H1N1), which shared high similarity with HuN EA-H1N1 virus; and blue indicates 2 human Eurasian avian-like influenza A(H1N1) isolates. Scale bars indicate nucleotide substitutions per site.

Complete sequences of HuN EA-H1N1 virus were 98.2%–99.3% identical in all 8 gene segments with influenza A/swine/Guangxi/BB1/2013(H1N1) virus, which was isolated in 2013 from a swine in Guangxi Province, China (*18*). Phylogenetic analysis of all genes showed that HuN EA-H1N1virus was a reassortment of EA-H1N1, A(H1N1)pdm09, and CS H1N1 viruses (Figure 2) (Technical Appendix Figure 3). HA, NA, and M genes of the virus shared highest similarity with those of 2 other human EA-H1N1 viruses in China: JS/1/11 EA-H1N1 and A/Hebei-Yuhua/SWL1250/2012 (HB/1250/12). The remaining 5 genes (polybasic [PB] 1 and 2, polymerase [PA], nucleoprotein [NP], and nonstructural protein [NS]) in HuN EA-H1N1 virus differed from those in the 2 other EA-H1N1 viruses: 4 internal genes (PB2, PB1, PA, and NP) clustered with A(H1N1)pdm09 virus (Figure 2, panel B) (Technical Appendix Figure 3), and the NS gene shared a common ancestor with A(H1N1)pdm09 virus and derived from CS H1N1 virus.

### Virulence and Replication in Mice

**Figure 3:**
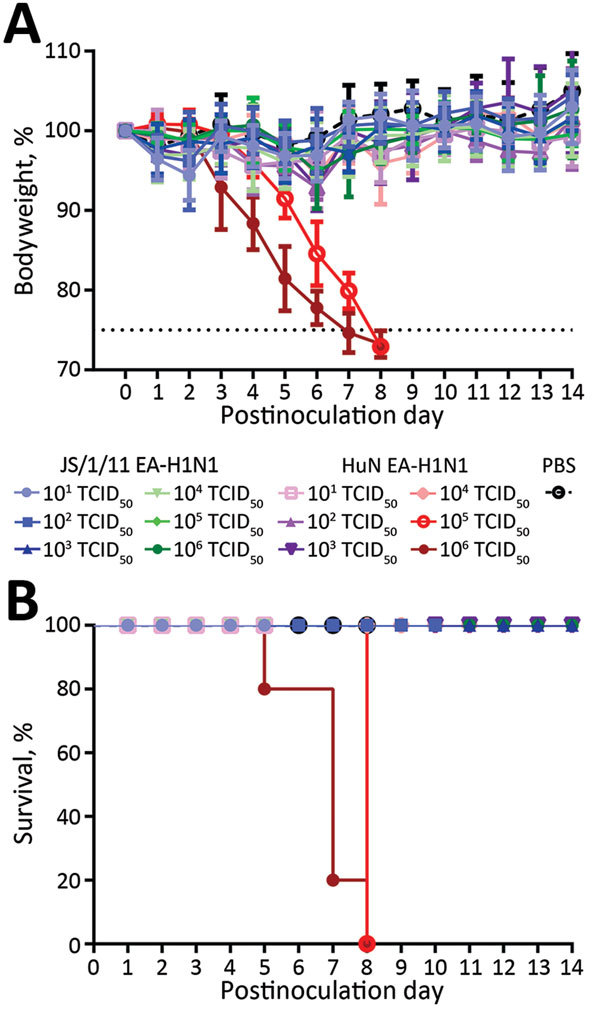
Illness (A) and death (B) among C57BL/6 mice inoculated with Eurasian avian-like influenza A/Jiangsu/1/2011 (JS/1/11 EA-H1N1) virus or Eurasian avian-like influenza A/Hunan/42443/2015 (HuN EA-H1N1) virus. Eight-to-ten week old female C57BL/6J mice (5/group) were inoculated intranasally with various 50% tissue culture infectious doses (TCID_50_) of JS/1/11 EA-H1N1 virus or HuN EA-H1N1 virus (in 50-μL of PBS) or with 50 μL mL of PBS (control group). A) Illness was assessed by weight changes over 14 days and is graphed as a percentage of the average weights on the day of inoculation (day 0). Average bodyweight changes ± SD are shown. Dotted horizontal line indicates a bodyweight loss of 75%. B) Death was investigated by using a survival curve. On postinoculation day 14, mice with a bodyweight loss of >25% and those who died naturally (i.e., not including those that were euthanized) were recorded as fatalities. PBS, phosphate-buffered saline.

To evaluate the virulence of JS/1/11 EA-H1N1 and HuN EA-H1N1 viruses, we first determined the MID_50_ (50% mouse infectious dose) and MLD_50_ (50% mouse lethal dose) for each virus by inoculating 3 groups of mice, respectively, with 10-fold serial dilutions (10^1^–10^6^ TCID_50_/50 μL) of JS/1/11 EA-H1N1 virus, HuN EA-H1N1 virus, or PBS. By 14 dpi, no JS/1/11 EA-H1N1 virus–inoculated mice had lost >10% of their bodyweight, and none had died, indicating an MLD_50_ of >10^6.5^ TCID_50_ for the virus (Figure 3, panel A). A similar trend in bodyweight loss was seen in mice inoculated with 10^1^–10^4^ TCID_50_ of HuN EA-H1N1 virus. However, at 8 dpi, mice inoculated with 10^5^ or 10^6^ TCID_50_ of HuN EA-H1N1 virus had lost >25% of their bodyweight (Figure 3, panel A), and by 14 dpi, all mice in these 2 groups (5 mice/group) had died, indicating an MLD_50_ value of 10^4.5^ TCID_50_ for the virus (Figure 3, panel B).

HI antibody testing showed that the MID_50_ for JS/1/11 EA-H1N1 virus was much higher than that for HuN EA-H1N1 virus (>10^4.7^ vs. 10^2.9^ TCID_50_, respectively) (Table 1). The cross-reactive antibody response for JS/1/11 EA-H1N1 and HuN EA-H1N1 indicated similar antigenicity (Table 1).

**Table 1:** Seroconversion in C57BL/6 mice inoculated with JS/1/11 EA-H1N1 and HuN EA-H1N1 viruses*

Virus and dose, log_10_ TCID_50_/50 μL	Hemagglutination inhibition titer†											MID_50_, log_10_ TCID_50_‡
	JS/1/11 EA-H1N1 virus antigen						HuN EA-H1N1 virus antigen					
Mouse 1	Mouse 2	Mouse 3	Mouse 4	Mouse 5	Mouse 1	Mouse 2	Mouse 3	Mouse 4	Mouse 5
JS/1/11 EA-H1N1												4.7
6	40	20	>80	40	40		>80	40	>80	40	40	
5	<10	40	20	20	40		10	>80	20	40	>80	
4	10	10	<10	<10	<10		20	<10	<10	10	<10	
3	<10	<10	<10	<10	<10		<10	<10	<10	<10	<10	
2	<10	<10	<10	<10	<10		<10	<10	<10	<10	<10	
1	<10	<10	<10	<10	<10		<10	<10	<10	<10	<10	
HuN EA-H1N1												2.9
6	ND	ND	ND	ND	ND		ND	ND	ND	ND	ND	
5	ND	ND	ND	ND	ND		ND	ND	ND	ND	ND	
4	>80	40	>80	>80	<10		>80	>80	>80	>80	<10	
3	>80	40	40	<10	20		>80	>80	80	<10	40	
2	<10	<10	<10	<10	<10		<10	<10	<10	<10	<10	
1	<10	<10	<10	<10	<10		<10	<10	<10	<10	10	
PBS	<10	<10	<10	<10	<10		<10	<10	<10	<10	<10	

*Serum samples were obtained from mice 14 d after inoculation. HuN EA-H1N1, Eurasian avian-like influenza A/Hunan/42443/2015(H1N1) virus; JS/1/11 EA-H1N1, Eurasian avian-like influenza A/Jiangsu/1/2011(H1N1) virus; MID_50_, mouse infectious dose; ND, not determined due to death of the animal (treated as positive for determination of the MID_50_); TCID_50_, 50% tissue culture infectious dose. †Titers <20 were regarded as negative for seroconversion. ‡MID_50_ was determined using the Spearman–Karber method (*19*).

**Figure 4:**
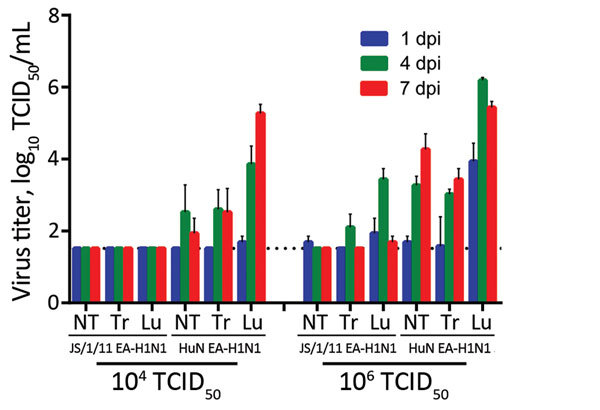
Replication of Eurasian avian-like influenza A/Jiangsu/1/2011 (JS/1/11 EA-H1N1) virus and Eurasian avian-like influenza A/Hunan/42443/2015 (HuN EA-H1N1) virus in the respiratory tracts of C57BL/6 mice. Eight- to ten-week-old female mice (3/group/time point) were inoculated intranasally with 50 μL of PBS containing 10^4^ or 10^6^ TCID_50_ of JS/1/11 EA-H1N1 or HuN EA-H1N1 virus. Mice from each group were euthanized at 1, 4, and 7 days postinoculation (dpi). Tissues from each animal were homogenized in 1 mL of PBS and then clarified by centrifugation, and virus titers in the supernatant were determined by TCID_50_ assay using MDCK cells. Results are the mean ± SD. NT, nasal turbinate; Tr, trachea; Lu, lung; TCID_50_, 50% tissue culture infectious dose. Dotted horizontal line indicates the detection limit.

We also investigated the tissue tropism and replication of HuN EA-H1N1 and JS/1/11 EA-H1N1 viruses in 2 groups of mice. Both viruses replicated in the respiratory tract, but infectivity was substantially divergent (Figure 4). At 1, 4, and 7 dpi, no virus was detected in any tissues of mice inoculated with 10^4^ TCID_50_ of JS/1/11 EA-H1N1 virus. However, at the same time points, virus was clearly present in the respiratory tract, including the nasal turbinates, trachea, and lungs, of mice inoculated with 10^4^ TCID_50_ of HuN EA-H1N1 virus. These findings were consistent with antibody responses in the mice (Table 1).

Mice inoculated with 10^6^ TCID_50_ of JS/1/11 EA-H1N1 virus had limited virus replication in nasal turbinates and tracheal tissues. However, virus was clearly present in lung tissues at 1 and 4 dpi, but titers decreased rapidly by 7 dpi (Figure 4). Compared with JS/1/11 EA-H1N1 virus–infected mice, those infected with HuN EA-H1N1 virus had substantially higher virus titers in nasal turbinates, trachea, and lungs (Figure 4) and occasionally in extrapulmonary organs, including the liver (10^2.0^ TCID_50_/mL) and kidney (10^1.8^ TCID_50_/mL) of 1 mouse. A high virus titer was persistent in the respiratory tracts of HuN EA-H1N1–infected mice. These findings were consistent with bodyweight and survival data (Figure 3).

### Serologic Investigation

We conducted a retrospective study to investigate the potential for human-to-human transmission of HuN EA-H1N1 virus, a reassortant EA-H1N1 virus. A total of 12 close contacts (4 family members and 8 healthcare workers) of the patient were included in this investigation. No family members reported influenza-like symptoms, but 3 healthcare workers showed signs and symptoms of illness. Whole-blood specimens from all close contacts were obtained for HI testing (*15*,*16*). An antibody response to HuN EA-H1N1 virus (HI titer of 40) was detected in only 1 contact, a doctor who exhibited signs of illness (Table 2). However, fever developed in this person on the same day she had contact with the patient, making it unlikely that her symptoms were caused by exposure to the patient. Furthermore, the positive HI antibody result for this contact may have been caused by a cross-reaction with A(H1N1)pdm09 virus (*11*,*20*). Thus, these findings indicate that human-to-human transmission of HuN EA-H1N1 virus did not occur.

**Table 2:** HI antibody titers to HuN EA-H1N1 virus and CA07 virus in serum collected from a HUN EA-H1N1–infected patient and his close contacts, Hunan Province, China, 2015*

Contact type and age, y	Contact date		Fever	Date of illness onset	Date of serum sample collection	HI titer	
	Initial	Final				HuN EA-H1N1	CA07
Doctor, 26	Unknown	Unknown	No	NA	Sep 21	5	40
Doctor, 28	Unknown	Unknown	No	NA	Sep 21	5	5
Nurse, 33	Unknown	Unknown	No	NA	Sep 21	20	40
Doctor, 36	Jul 2	Jul 11	Yes	Jul 11	Sep 21	5	5
Doctor, 47	Jul 13	Jul 13	Yes	Jul 13	Sep 28	40	80
Doctor, 39	Jul 13	Jul 13	No	NA	Sep 28	5	160
Doctor, 45	Jul 2	Jul 2	Yes	Jul 2	Sep 28	5	40
Nurse, 38	Jul 2	Jul 13	No	NA	Sep 28	5	40
Parent, 43	Jun 30	Jul 1	No	NA	Sep 29	20	40
Parent, 42	Jun 30	Jul 1	No	NA	Sep 29	5	5
Grandparent, 67	Jun 30	Jul 1	No	NA	Sep 29	5	40
Grandparent, 63	Jun 30	Jul 1	No	NA	Sep 29	5	5
Patient, 2	NA	NA	Yes	Jun 30	Sep 29	80	160

*CA07, A/California/07/2009 [A( H1N1)pdm09 virus; HI, hemagglutination inhibition; HuN EA-H1N1, Eurasian avian-like influenza A/Hunan/42443/2015(H1N1) virus; NA, not available.

## Discussion

We identified a reassortant EA-H1N1 virus, HuN EA-H1N1, in a child in China with severe pneumonia. HA, NA, and M genes of the HuN EA-H1N1virus were derived from EA-H1N1 viruses; PB2, PB1, PA, and NP genes were derived from A(H1N1)pdm09 virus; and NS gene was derived from CS H1N1 virus. Our virulence studies in C57BL/6 mice showed that HuN EA-H1N1 virus exhibited greater virulence than JS/1/11 EA-H1N1 virus, which had been previously isolated from a child with fatal infection (Figure 3; Table 1). The full genome of another EA-H1N1 virus, HB/1250/12, which was isolated from a patient with mild illness, shared 98.9–99.6% nt identity with JS/1/11 EA-H1N1 virus, and these viruses caused similar illness and fatality rates in mice (data not shown). These findings suggest that the internal genes of HuN EA-H1N1 virus could be one a cause of the severe clinical syndrome seen in the HuN EA-H1N1 virus–infected child. Furthermore, the presence of renal disease and a history of long-term steroid treatment could have been major contributors to the death of the JS/1/11 EA-H1N1 virus–infected child (*10*).

A previous study showed that EA-H1N1 SIVs preferentially bind to human-type receptors, and some of the tested viruses were transmitted to ferrets by airborne droplets (*12*). That study concluded that EA-H1N1 SIVs have the potential to transmit efficiently and to cause a human influenza pandemic. We report a case of human infection with a reassortant EA-H1N1 swine influenza virus. Four internal genes of the virus were derived from A(H1N1)pdm09 virus. The 190D aa and 225E aa (H3 numbering) in the HA protein of the HuN EA-H1N1 virus suggested preferential binding to the SAα-2,6-Gal receptor. Although human-to-human transmission was not detected in our study, EA-H1N1 viruses have been reported to transmit efficiently via respiratory droplets in the ferret model (*12*). In addition, EA-H1N1 virus gene segments (NA and M genes) have been reported to contribute to the efficient respiratory droplet transmission of A(H1N1)pdm09 viruses in the ferret model (*21*). Our studies in mice also showed that infectivity and virulence were increased in reassortants of EA-H1N1 virus and A(H1N1)pdm09 virus. Thus, given the prevalence of novel EA-H1N1 viruses in pigs and their potential transmissibility to and pathogenicity in humans, enhanced influenza surveillance should be instituted among swine and humans.

Our study had several limitations. First, potential sources of infection for the HuN EA-H1N1 virus–infected patient were not identified. We collected 9 swab samples from pigs raised on the premises of the patient’s home, which was in a rural location, but EA-H1N1 virus was not isolated. Second, despite the high similarity of HA (98.9%) and NA (97.7%) genes from JS/1/11 EA-H1N1 and HuN EA-H1N1 viruses (online Technical Appendix Table), we could not conclude that the A(H1N1)pdm09 virus–derived internal genes were associated with the high virulence of HuN EA-H1N1 virus and the patient’s severe clinical syndrome. More investigations are needed to verify the virulence factor of HuN EA-H1N1 virus. Third, because an acute-phase serum sample was not available, we could not determine whether a doctor who exhibited signs of illness became infected before or after exposure to the patient; however, the doctor had a fever the same day she had contact with the patient.

In conclusion, EA-H1N1 swine influenza viruses occasionally infect humans. We report on a novel EA-H1N1 virus reassortant, HuN EA-H1N1 virus, which was isolated from a boy in China with severe pneumonia. The virus contained 2 surface genes from an EA-H1N1 virus and 4 internal genes from A(H1N1)pdm09 virus. Compared with JS/1/11 EA-H1N1 virus, the reassortant virus exhibited higher infectivity, virulence, and replication in C57BL/6J mice, demonstrating the need for further evaluation of HuN EA-H1N1 virus to assess the threat it poses to public health. Our results indicate the need for heightened surveillance.

Technical Appendix Supporting data on the patient’s chest radiographs, microbe screening, amino acids differences, and phylogenetic analysis of the investigated influenza viruses.
