# Supplementary material for: Reassortant Eurasian Avian-Like Influenza A(H1N1) Virus from a Severely Ill Child, Hunan Province, China, 2015
Source: Emerg Infect Dis. 2016 Nov;22(11):1930–6. doi: 10.3201/eid2211.160181 (PMC5088044; doi:10.3201/eid2211.160181)
Supplement: Technical Appendix — Supporting data on the patient’s chest radiographs, microbe screening, amino acids differences, and phylogenetic analysis of the investigated influenza viruses. [file 16-0181-Techapp-s1.pdf]

Article DOI: http://dx.doi.org/10.3201/eid2211.160181

Reassortant Eurasian Avian-Like Influenza A(H1N1) Virus from a Severely Ill Child, Hunan Province, China, 2015

Technical Appendix

Technical Appendix Table. Amino acid differences on HA and NA proteins between A/Hunan/42443/2015 and A/Jiangsu/1/2011 (JS1) viruses

Proteins	HA (H1 numbering)						NA													
Sites number	56	236	259	260	355	398	12	16	45	46	76	79	86	255	286	289	314	321	340	382
A/Hunan/42443/2015	S	D	K	K	Q	F	S	A	Q	S	T	V	A	I	N	I	I	I	P	R
A/Jiangsu/1/2011	N	H	N	E	R	C	L	T	K	P	A	A	V	V	S	T	M	V	S	K

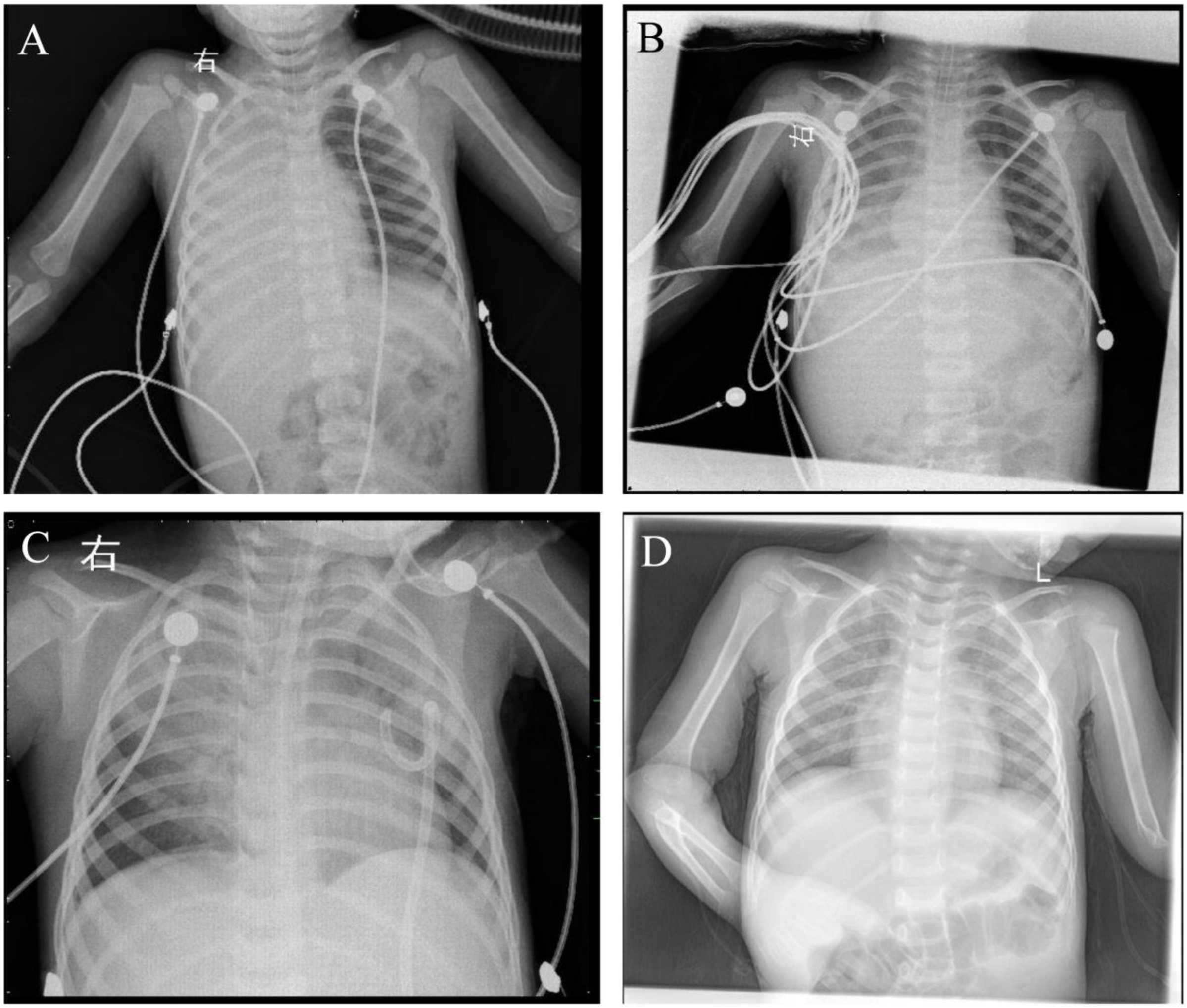

Technical Appendix Figure 1. Chest radiographs of the investigated patient on day 2 (A), day 6 (B), day 16 (C), and day 24 (D) after illness onset. A) A right pleural effusion and a collapsed lower right lung were found. B) A small amount of right pleural effusion and patchy shadow was observed in the lower right lung. C) Shadows were observed in bilateral upper lung and part of the lower lung lesions disappeared. D) No obvious abnormalities of the cardiophrenic space was found.

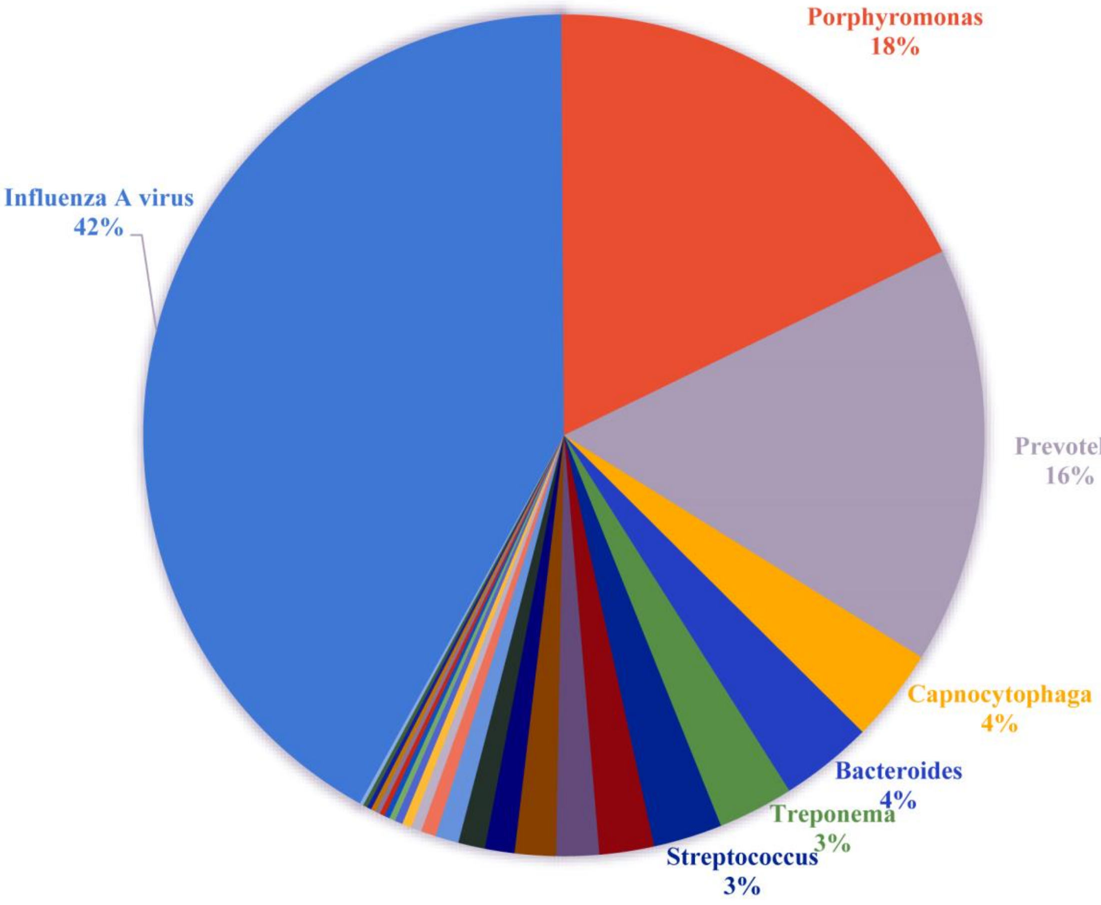

Technical Appendix Figure 2. Pie chart of reads assigned to microbes. The influenza A virus accounted for the majority of reads (41.9%) from the sample, while *Porphyromonas* and *Prevotella* species accounted for 17.9% and 16.0%, respectively.

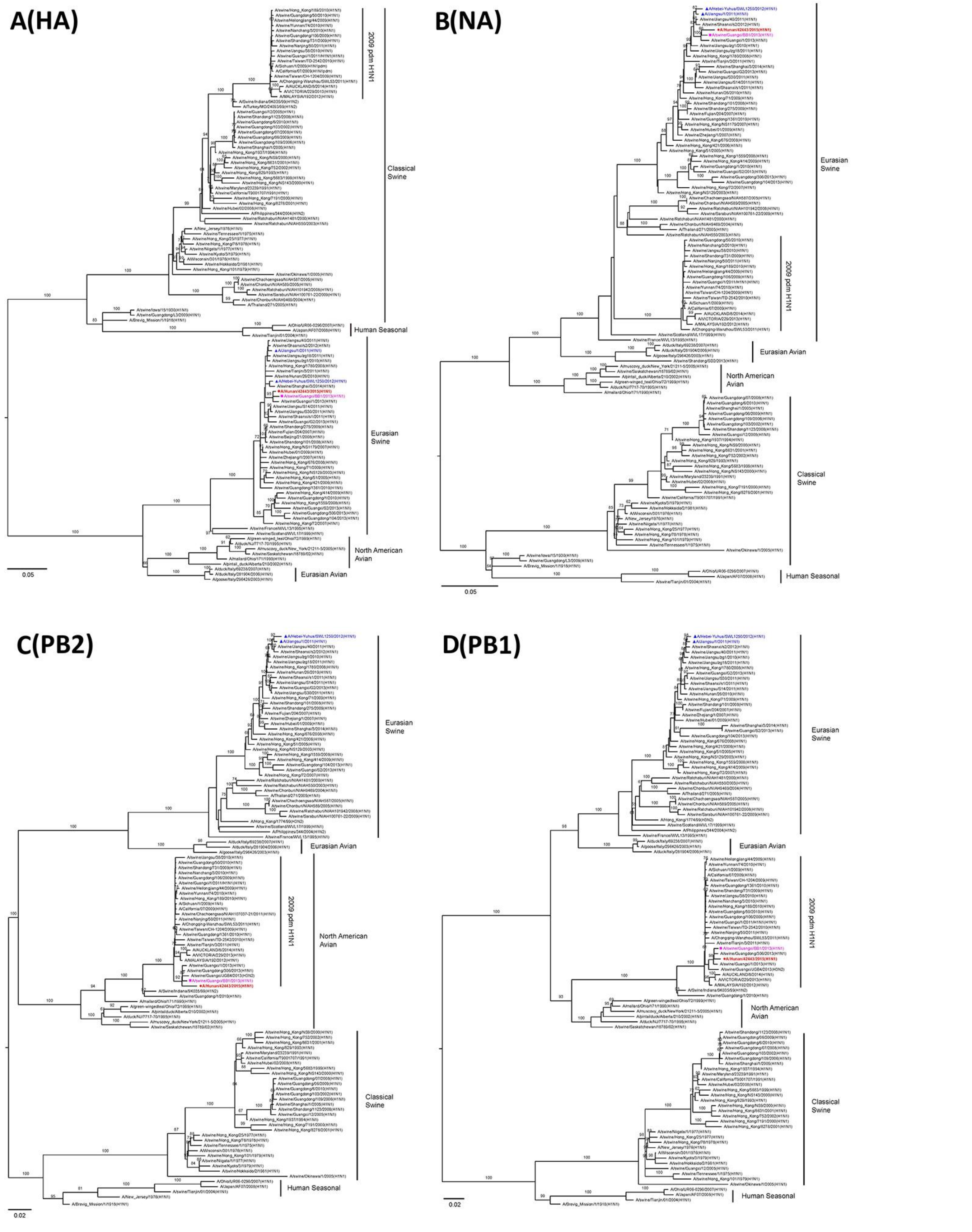
A(HA)
2009 pdm H1N1
Classical Swine
Human Seasonal
Eurasian Swine
North American Avian
Eurasian Avian
0.05
B(NA)
Eurasian Swine
2009 pdm H1N1
Eurasian Avian
North American Avian
Classical Swine
Human Seasonal
0.05
C(PB2)
Eurasian Swine
Eurasian Avian
2009 pdm H1N1
North American Avian
Classical Swine
Human Seasonal
0.02
D(PB1)
Eurasian Swine
Eurasian Avian
2009 pdm H1N1
North American Avian
Classical Swine
Human Seasonal
0.02

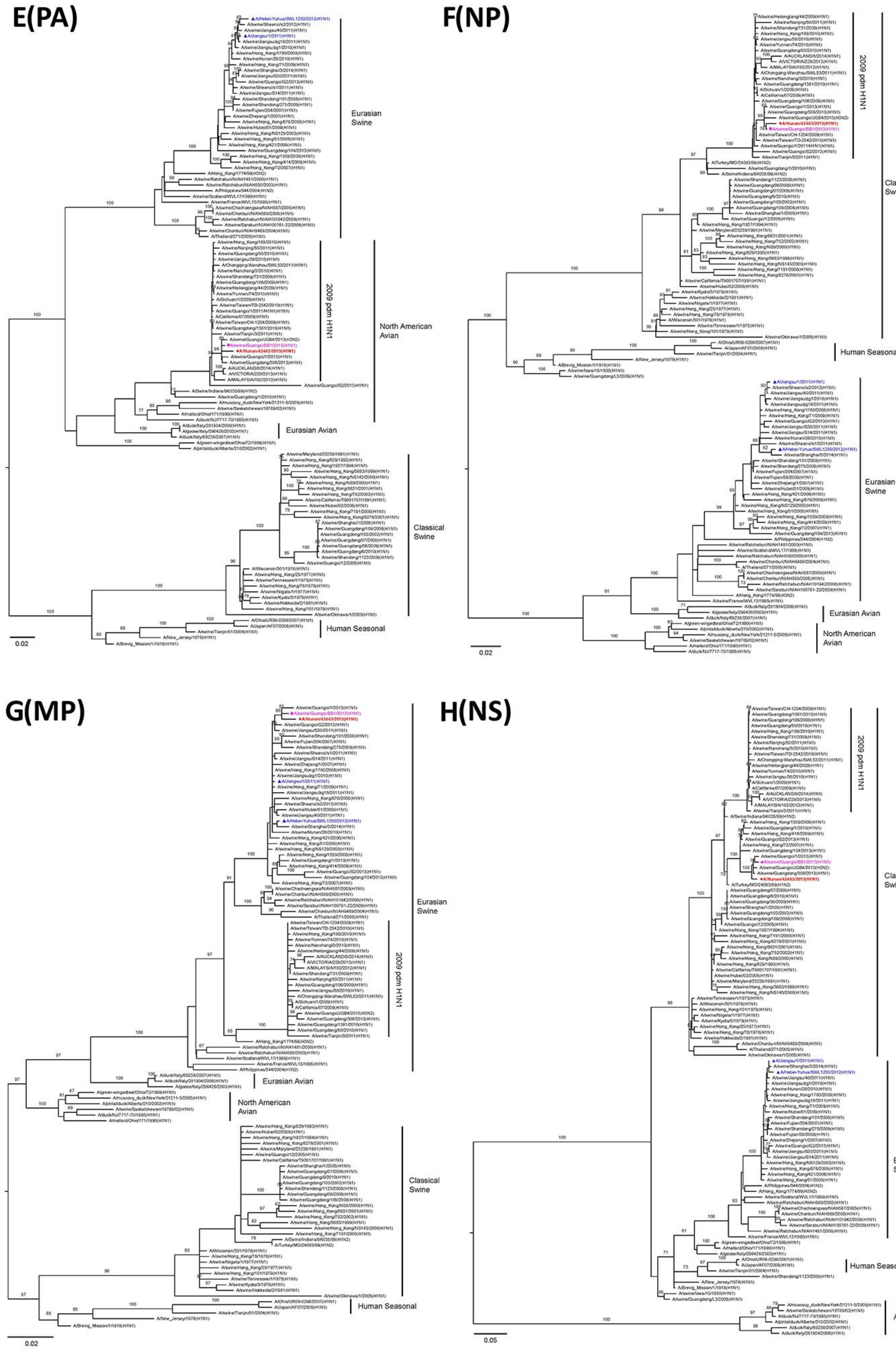
E(PA)
Eurasian Swine
2009 pdm H1N1
North American Avian
Eurasian Avian
Classical Swine
Human Seasonal
0.02
F(NP)
2009 pdm H1N1
Classical Swine
Human Seasonal
Eurasian Swine
Eurasian Avian
North American Avian
0.02
G(MP)
Eurasian Swine
2009 pdm H1N1
Eurasian Avian
North American Avian
Classical Swine
Human Seasonal
0.02
H(NS)
2009 pdm H1N1
Classical Swine
Eurasian Swine
Human Seasonal
Avian
0.05

Technical Appendix Figure 3 (following pages). Phylogenetic analysis of all eight genes on A/Hunan/42443/2015 (HuN) viruses. Phylogenetic trees of HA (A), NA (B), PB2 (C), PB1 (D), PA (E), NP (F), MP (G), and NS (H) genes. The virus reported in our study (A/Hunan/42443/2015(H1N1), HuN) is highlighted in red, and the EA viruses isolated from mainland China previously are in blue. The virus (A/swine/Guangxi/BB1/2013 (BB1, H1N1)), which shared the highest similarities with HuN virus, is included for analysis in each tree. A solid circle, square, and triangle are shown at the beginning of the HuN, BB1, and 2 human EA H1N1 viruses, respectively.
